# Fibronectin and vitronectin alleviate adipose-derived stem cells senescence during long-term culture through the AKT/MDM2/P53 pathway

**DOI:** 10.1038/s41598-024-65339-z

**Published:** 2024-06-20

**Authors:** Patcharapa Tragoonlugkana, Chatchai Pruksapong, Pawared Ontong, Witchayapon Kamprom, Aungkura Supokawej

**Affiliations:** 1 Department of Clinical Microscopy, Faculty of Medical Technology, Mahidol University, 999 Phutthamonthon Sai 4, Salaya, Phutthamonthon, Nakhon Pathom, 73170 Thailand; 2 Department of Surgery, Phramongkutklao Hospital and Phramongkutklao College of Medicine, Bangkok, 10400 Thailand; 3 Department of Community Medical Technology, Faculty of Medical Technology, Mahidol University, Nakhon Pathom, 73170 Thailand; 4 Department of Clinical Microbiology and Applied Technology, Faculty of Medical Technology, Mahidol University, Nakhon Pathom, 73170 Thailand

**Keywords:** Adipose-derived stem cells, Cell therapy, Good manufacturing practice, Cell culture, Replicative senescence, Fibronectin, Vitronectin, Senescence, Ageing, Mesenchymal stem cells, Stem-cell research

## Abstract

Cellular senescence plays a role in the development of aging-associated degenerative diseases. Cell therapy is recognized as a candidate treatment for degenerative diseases. To achieve the goal of cell therapy, the quality and good characteristics of cells are concerned. Cell expansion relies on two-dimensional culture, which leads to replicative senescence of expanded cells. This study aimed to investigate the effect of cell culture surface modification using fibronectin (FN) and vitronectin (VN) in adipose-derived stem cells (ADSCs) during long-term expansion. Our results showed that ADSCs cultured in FN and VN coatings significantly enhanced adhesion, proliferation, and slow progression of cellular senescence as indicated by lower SA-β-gal activities and decreased expression levels of genes including p16, p21, and p53. The upregulation of integrin α5 and αv genes influences phosphatidylinositol 4,5-bisphosphate 3-kinase (PI3K), and AKT proteins. FN and VN coatings upregulated AKT and MDM2 leading to p53 degradation. Additionally, MDM2 inhibition by Nutlin-3a markedly elevated p53 and p21 expression, increased cellular senescence, and induced the expression of inflammatory molecules including HMGB1 and IL-6. The understanding of FN and VN coating surface influencing ADSCs, especially senescence characteristics, offers a promising and practical point for the cultivation of ADSCs for future use in cell-based therapies.

## Introduction

Senescence, a key process that causes aging, was first discovered by Hayflick and colleagues, who reported that fibroblasts during in vitro display phenotypic changes and stop growing^1^. Based on their study, the Hayflick limit was established, i.e., every cell type has its own limitation of growth^2^. The senescent characteristics during the aging process have been determined and include telomere shortening, the expression of cell-cycle inhibitor protein, the upregulation of senescent-associated genes, and changes in signaling pathways involved in cell growth and differentiation^3^. The accumulation of senescent cells leads to tissue and organ dysfunctions, which are associated with several degenerative diseases. Currently, treatment for degenerative diseases is challenging, but cell therapy has shown promising results in this regard. Cell therapy is recognized as a treatment approach for tissue regeneration in which living cells are used to treat, prevent, or cure diseases or injuries. The type of cell used in cell therapy depends on the condition being treated, e.g., diseases of the immune, blood, and stem cells^4^. Mesenchymal stem/stromal cells (MSCs) have shown promising therapeutic outcomes in the field of regenerative medicine^5^. Adipose-derived stem cells (ADSCs) are an alternative source of MSCs that can be isolated from fat tissue by lipoaspiration or fat biopsy. They showed high potential in rapid proliferation and immunosuppressive capabilities, allowing modulation of immune responses and potentially minimizing immune rejection in cell therapy applications^5,6^.

For clinical use of ADSCs, cell processing is performed under restricted regulation according to good manufacturing process (GMP)^7^. Many factors within culture conditions can influence cellular properties, including culture medium, temperature, physical support, pH, and culture vessels. These factors should be considered and validated for continuing of reproducibility and traceability. In vitro cell expansion influences the properties of ADSCs demonstrating by replicative senescent of ADSCs during culture prolongation. The extensive growth of ADSCs to reach the therapeutic dose slowed the progression of cell growth and prolonged the population doubling time. The morphological alteration in abnormalities of the nuclear morphology^8^ and cell size is indicated by the large, flattened cytoplasm with granules^9^. In addition, aging ADSCs increased the expression of senescence-associated β-galactosidase (SA-β-gal) and secreted proinflammatory factors known as senescence-associated secretory phenotype (SASP) that influence the surrounding microenvironment through cell or tissue aging^9^. The unsatisfactory outcome of the clinical study was raised as a major concern, and the quality of ADSCs was shown to impact the treatment outcome, leading to unsuccessful results^10^. However, replicative senescence can be prevented by optimizing culture conditions, genetic modification, antioxidant supplementation, and senescence inhibitors^11–14^. These methods can be used individually or in combination, depending on the specific cell culture system and desired outcomes.

Growing cells in culture vessels coated with adhesion molecules aimed to increase cell adhesion^15,16^. Typically, adhesion molecules, such as collagen, fibronectin (FN), laminin, and vitronectin (VN), are used as extracellular matrix (ECM) coatings^17–19^, but these are expensive and mostly obtained from animal sources. FN and VN are found in fresh human plasma and can be easily purified^20,21^. FN can attach to cells through specific binding sites and integrin receptors on the cell surface via the cell-binding domain, particularly prominent on α5β1 integrin through the arginine-glycine-aspartic acid (RGD) sequence in tenth fibronectin type III^22^. Besides, VN binds to cells through its interaction with integrin receptors on the cell surface. Specifically, it binds to αVβ3 and αVβ5 integrin through the RGD sequence and promotes cell adhesion, spreading, and migration^23^. A recent study demonstrated that FN and VN play crucial roles in regulating cell cycle progression and apoptosis. FN induces cell cycle arrest by upregulating p21 and can modulate cell survival or apoptosis depending on cellular context. FN signaling through integrins activating prosurvival pathways like PI3K-AKT to inhibit apoptosis^24,25^. Similarly, VN has dual roles in cell survival and apoptosis regulation by influencing cell cycle progression and apoptosis through the PI3K-AKT pathway activated by integrin receptors. It has also been associated with apoptosis-inducing processes like Anoikis, which is triggered by loss of cell adhesion^26,27^. Additionally, FN and VN have the potential to prevent cellular senescence by modulating the PI3K/AKT signaling pathway^24–27^. Interestingly, coating with these human-derived proteins can improve the cell expansion process, enhance cell survival, and boost cell growth performance. However, there is a lack of clarity regarding its role in cellular senescence.

Currently, the process of cell processing especially ADSCs needs cell expansion steps to provide a vast number of therapeutic cells. The standard practice for this process is under development and shows variability. To meet the good quality of ADSCs, the optimized cell culture surface modification using adhesion molecules, including FN and VN might be a suitable process to reduce the senescent cells. In this study, we investigated the senescent characteristics of ADSCs during long-term expansion. To explore the impact of these adhesion molecules on cellular senescence, the aging-related cellular signaling pathway was investigated. The use of adhesion molecules is expected to promote cell adhesion, improve proliferation, and reduce replicative senescence, thereby enhancing the overall MSCs culture process. The finding of this study will support the role of adhesion molecules in therapeutic cell growth, with the encouragement of a good protocol for cell culture in a clinical setting.

## Results

### ADSCs cultured with FN and VN coating enhanced adhesion and proliferation

**Figure 1:**
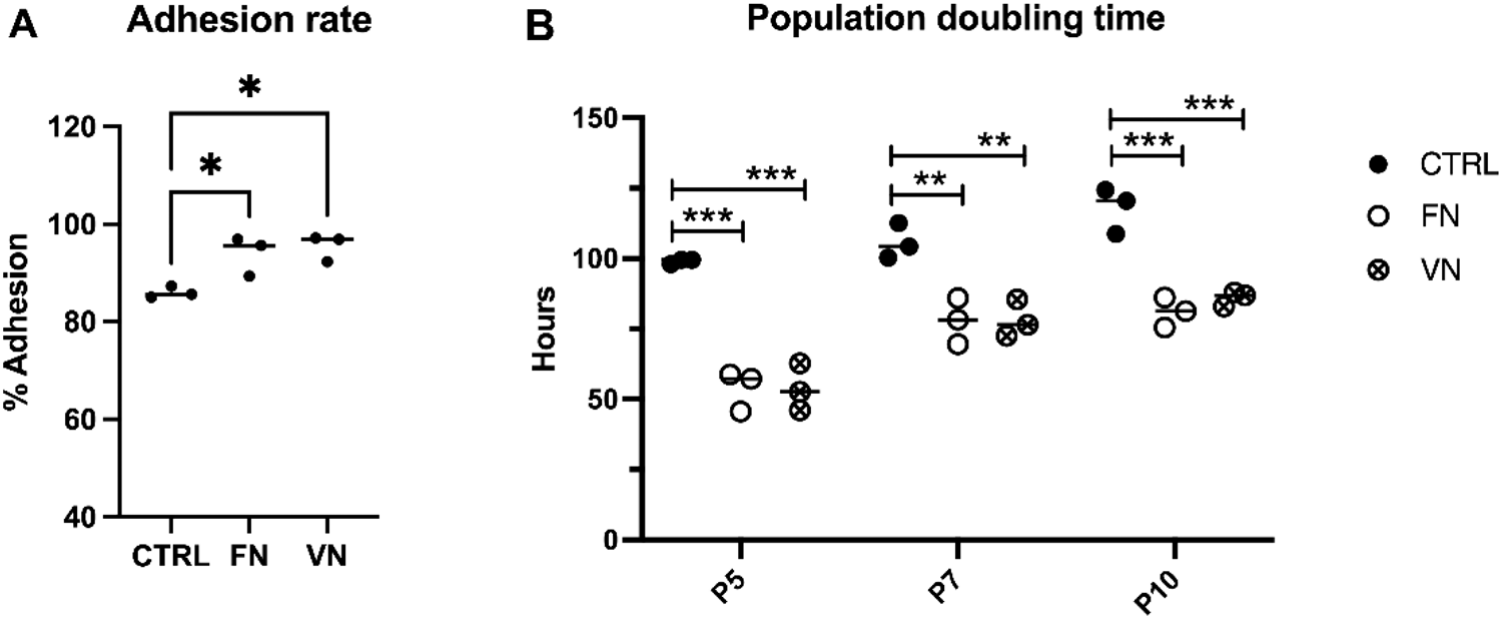
The effect of FN and VN coatings on ADSCs cultured. (**A**) Percent cell adhesion rate of ADSCs after culture on adhesion molecules including FN and VN for 12 h compared to the CTRL. The data indicated the means ± SD from three individual experiments, *p < 0.05. (**B**) Each population doubling time (PDT) was analyzed in P5, P7, and P10, respectively. These passages exhibited significantly lower PDT values for the FN and VN coatings compared with CTRL. Error bars represent the means ± SD, n = 3; **p < 0.01, ***p < 0.001; *P* passage.

To determine the cell properties of ADSCs culture in FN and VN coating, adhesion rate and PDT was performed. The various concentrations of FN and VN were investigated to determine the appropriate concentration for this study (Supplementary Table 1). The coated surface with the lowest concentration of FN and VN which promoted adhesion and PDT was employed in this study. The adhesion rates of ADSCs cultured in FN and VN were investigated after cell seeding for 12 h. The number of adherence cells was counted and presented as the percentage of the seeding cell number. Our results showed that the adhesion rates of cells in wells coated with FN (91.46% ± 3.60%) and VN (93.72% ± 3.60%) were significantly higher than the control group (CTRL) (83.22% ± 3.62) (*p* < 0.05) (Fig. 1A). The time interval spent in response to cell-cycle activity of ADSCs was determined using PDT assay. The PDT values of ADSCs growth in FN- and VN-coated at P5, P7, and P10 are presented in Fig. 1B which was significantly shorter than the control group in the indicated passages.

### ADSCs cultured with FN and VN coating slows the progression of cellular senescence

**Figure 2:**
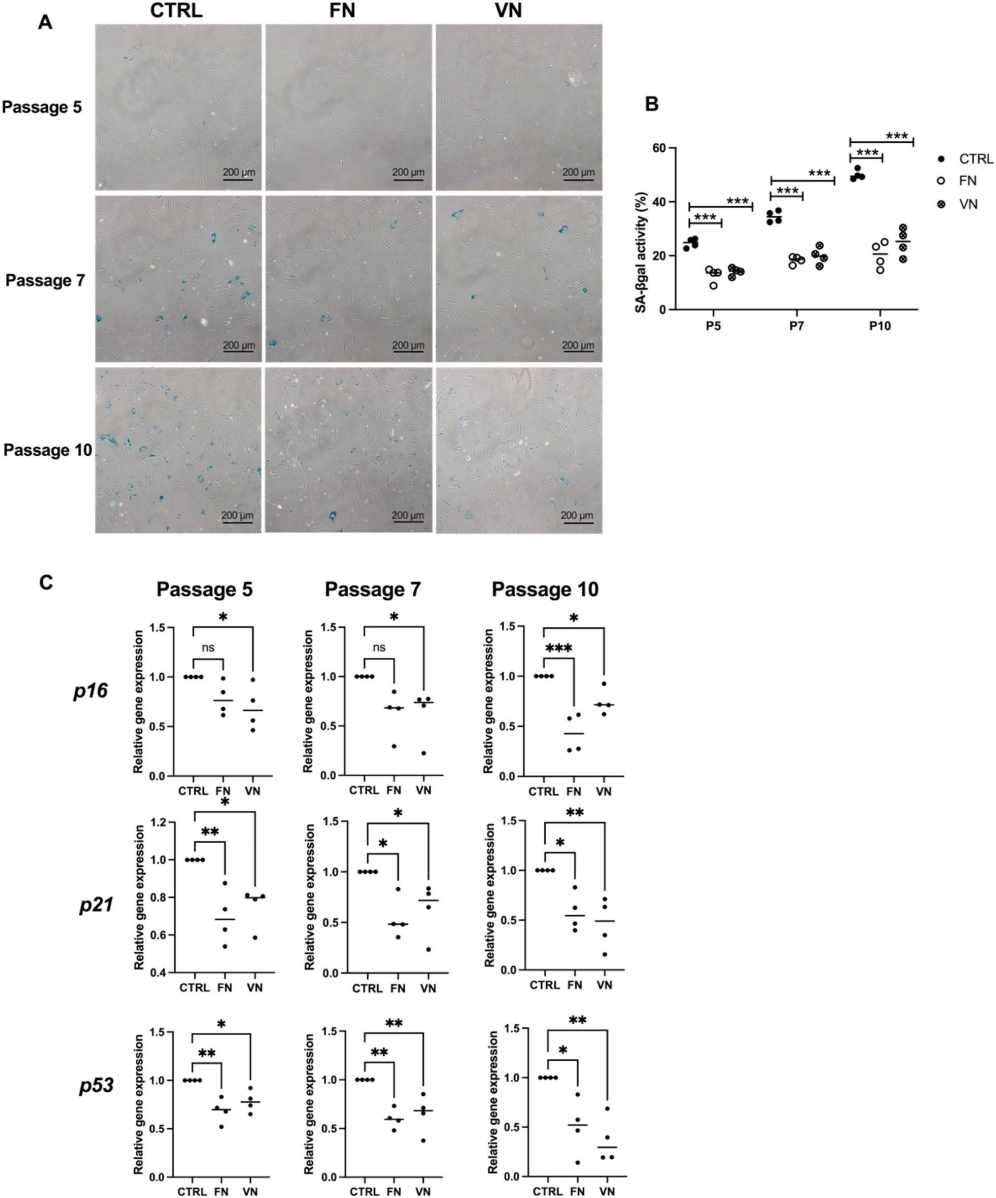
The effect of FN and VN coating on ADSCs senescence after serial passage (P5, P7, and P10). (**A**) SA-β-gal–stained positive cells of ADSCs cultured with FN and VN coating at P5, P7, and P10 showed blue staining (Scale bar = 200 μm). (**B**) The number of senescent cells of ADSCs at P5, P7, and P10 were counted and calculated as a percentage of SA-β-gal activity compared with the control. The data is presented as the means ± SD (n = 4). ***p < 0.001 *vs.* CTRL. (**C**) The mRNA expression levels of *p16, p21,* and *p53* of ADSCs at P5, P7, and P10. The data shows the means ± SD from four individual experiments. *p < 0.05 and **p < 0.01 *vs.* CTRL.

ADSCs cultured with the conventional method (control) and adhesion molecules at P5, P7, and P10 were determined for the senescence cells using SA-β-gal staining (Fig. 2A). The number of SA-β-gal–stained cells was counted and presented in percentage. ADSCs in the control showed increased SA-β-gal positive cells at P5, P7, and P10 in a passage-dependent manner. ADSCs cultured with FN coating at P5, P7, and P10 exhibited 12.78 ± 2.66, 18.33 ± 1.41, and 20.24 ± 2.82 numbers of SA-β-gal–stained cells, respectively. Similarly, ADSCs cultured with VN coating at P5, P7, and P10 showed 14.05 ± 1.48, 19.89 ± 3.18, and 24.92 ± 5.11 numbers of SA-β-gal positive cells, respectively (Fig. 2B). Furthermore, the hallmarks of cellular senescence, including p16 and p53 that play essential functions in senescence-associated cell-cycle arrest, were evaluated. The expression of *p16* in ADSCs (P5, P7, and P10) cultured with FN and VN coatings was lower than that in the control group. Besides, the expressional levels of *p21* and *p53* in ADSCs cultured with adhesion molecules coating were significantly decreased in the indicated passages compared to the control group (Fig. 2C).

### Alteration of AKT/MDM2/p53 in ADSCs cultured with FN and VN mediated by integrin α5 and αv

**Figure 3:**
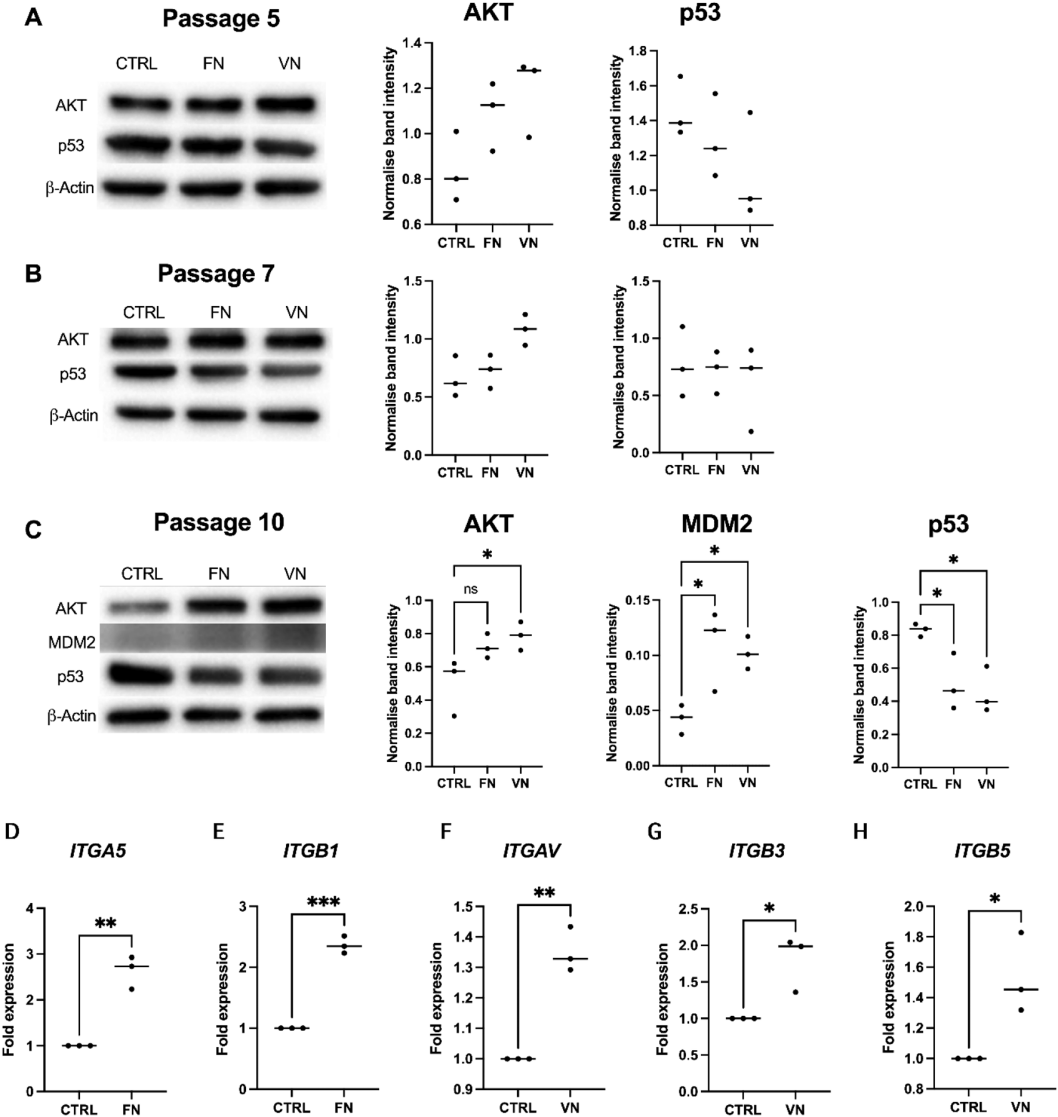
The expression of integrin α5 and αV in ADSCs cultured with FN and VN mediated AKT activation, which enhanced MDM2 and p53 degradation. The presence of AKT/MDM2/p53 signaling pathway was detected by western blotting assay and the representative quantitative of protein expression of ADSCs cultured with FN and VN coatings in passage (**A**) 5, (**B**) 7, and (**C**) 10. Quantifying of the mRNA expression levels of (**D**) *ITGA5* and (**E**) *ITGB1* in ADSCs cultured with FN, and (**F**) *ITGAV*, (**G**) *ITGB3*, and (**H**) *ITGB*5 in ADSCs cultured with VN. The expression of each gene (D-H) was presented as fold expression of control. The data show the means ± SD from three individual experiments. *p < 0.05 and **p < 0.01 *vs.* CTRL.

The expressions of AKT, MDM2, and P53 were investigated in ADSCs at P5, P7, and P10 to determine AKT/MDM2/p53 pathways involved in cellular senescence during in vitro expansion of ADSCs (Fig. 3A–C). ADSCs cultured with FN and VN coatings resulted in an increase in AKT protein, particularly in P10 (Fig. 3C). In addition, both FN and VN coatings were found to reduce p53 expression, which is a tumor-suppressor protein involved in cell-cycle arrest, senescence, and apoptosis. The expression of p53 was significantly decreased in ADSCs cultured with FN and VN at P10 (Fig. 3C). Moreover, MDM2 expression was also observed in ADSCs at P10 which higher in ADSCs cultured with FN and VN coatings than in the control (Fig. 3C). Besides, the expressions of integrin α5 and αv mediated AKT activation which enhancing p53 degradation. Herein, we investigated alterations in the gene expression of integrin subunits α5 (*ITGA5*) and β1 (*ITGB1*) in ADSCs cultured with FN. The gene expression of integrin subunits αv (*ITGAV*), β3 (*ITGB3*), and β5 (*ITGB5*) was also observed in ADSCs cultured with VN. As shown in Figs. 3D and E, the expression of *ITGA5* and *ITGB1* in ADSCs cultured with FN coating showed a significant increase compared to the control group. Moreover, the expressions of *ITGAV*, *ITGB3*, and *ITGB5* (Fig. 3F–H) were upregulated in ADSCs cultured with VN coating. Furthermore, gene expression of *ITGAV, ITGB3*, and *ITGB5* in ADSCs cultured with FN coating and *ITGA5* and *ITGB1* in ADSCs cultured with VN coating were examined and were not different from the control (Supplementary Fig. S1).

### The alteration of senescent-associated protein in ADSCs through AKT/MDM2/p53 pathway

**Figure 4:**
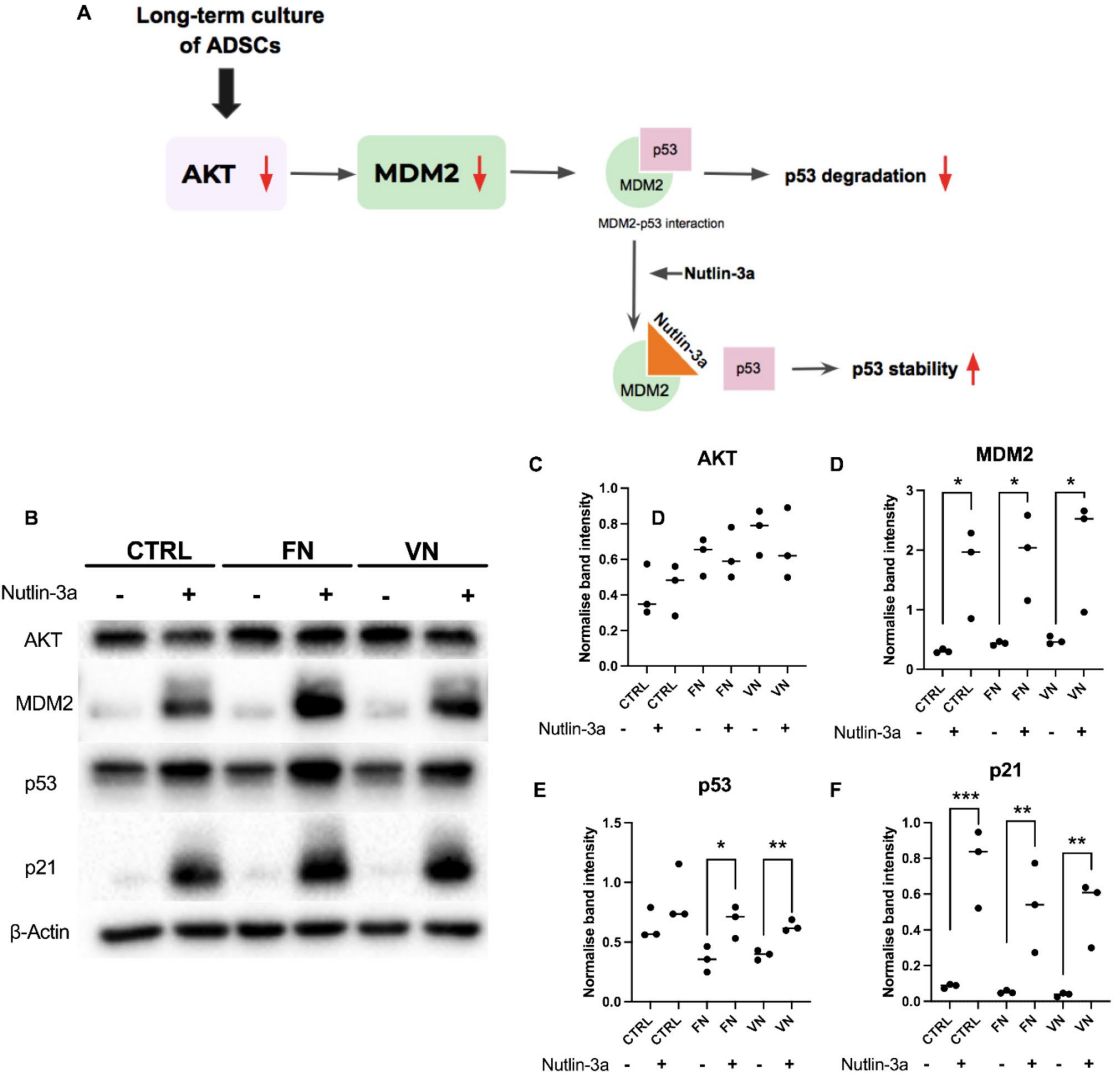
The regulation of cell senescence through AKT/MDM2/p53 pathway was determined. (**A**) The process of regulating cellular aging through the AKT/MDM2/p53 pathway. (**B**) The protein expression levels of AKT, MDM2, p53, and p21 in ADSCs, cultured with and without FN and VN coatings, were assessed after treatment with/without 10 μM Nutlin-3a at 48 h using Western blot analysis. β-Actin served as the internal control. Representative quantitative of protein expression (**C**) AKT, (**D**) MDM2, (**E**) p53, and (**F**) p21. The data showed the means ± SD from three individual experiments. *p < 0.05 and **p < 0.01 *vs.* CTRL.

To investigate the regulation of cell senescence through AKT/MDM2/p53, ADSCs cultured without and with FN and VN coatings were treated with Nutlin-3a which is an MDM2 inhibitor, that significantly suppresses p53 function by disrupting the p53–MDM2 interaction (Fig. 4A), Nutlin-3a was added to ADSCs culture at P10 to determine whether AKT regulated p53 expression through MDM2. The expression of AKT in ADSCs from control, FN, and VN coating was similar with each ADSCs treated with Nutlin-3a. (Fig. 4B,C). The MDM2 expression was higher in ADSCs (CTRL, FN, VN) treated with Nutlin-3a, which showed significant differences with non-Nutlin-3a-treated across all groups (Fig. 4B,D). The expression of MDM2 between the CTRL, FN, and VN (without Nutlin-3a) in P10 revealed significantly increased MDM2 expression in FN and VN coating (Supplementary Fig. S2). According to the regulatory role of MDM2 to p53, all groups treated with Nutlin-3a showed higher p53 expression (Fig. 4B,E). The expression of CDK inhibitor, p21, which is downstream of p53 and involved in cellular senescence, was significantly increased in ADSCs treated with MDM2 inhibitor (Fig. 4B,F). Taken together, ADSCs senescent occurring was controlled by MDM2-p53 interaction which subsequently affect p21 expression.

### The senescent phenotype in ADSCs influenced by AKT/MDM2/p53 pathway

**Figure 5:**
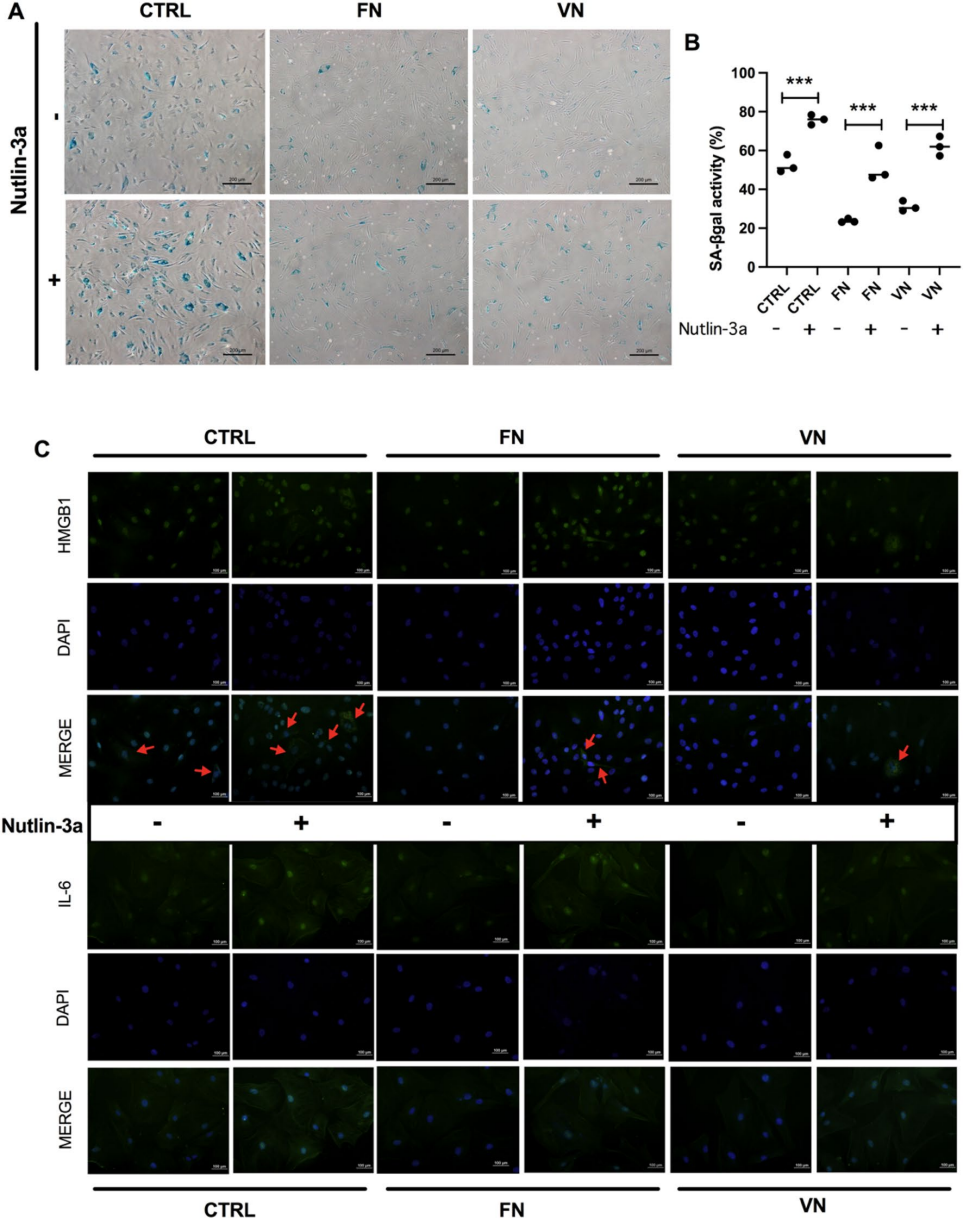
After 48 h, the assessment of the senescence phenotype was conducted on ADSCs at P10. ADSCs were cultured with the FN and VN coating followed by Nutlin-3a. (**A**) Representative images of the SA-β-gal positive cells of ADSCs which showed blue color (Scale bar = 200 μm). (**B**) Quantification of SA-β-gal positive cells, the number of senescent cells of ADSCs at P10 were counted and calculated as the percentage of SA-β-gal activity compared with the CTRL. The data is presented as the means ± SD (n = 3). ***p < 0.001 *vs.* CTRL. (**C**) Immunostaining revealed that treatment with Nutlin-3a can enhance the expression of HMGB1 from nucleus to cytoplasm (red arrows) and increase the secretion of IL-6. All scale bars represent 100 µm.

To demonstrate whether MDM2-p53 influences the senescent characteristic, ADSCs in the control group and cultured with FN and VN coatings were treated with Nutlin-3a. SA-β-gal staining and immunofluorescence analysis were applied. The SA-β-gal staining of ADSCs in control and FN, VN coatings revealed lower number of SA-β-gal positive cells than Nutlin-3a treated cells (Fig. 5A). The percentage of SA-β-gal was significantly higher in ADSCs treated with Nutlin-3a across all groups (Fig. 5B). Remarkably, High mobility group box 1 (HMGB1) and interleukin-6 (IL-6) are implicated in senescence by promoting inflammation and SASP. Consequently, the study of HMGB1 and IL-6 expression using immunofluorescence was performed. Immunostaining revealed that cells treated with Nutlin-3a showed HMGB1 localized in the cytoplasm compared with the non-Nutlin-3a-treated cells in which HMGB1 localized in the nucleus (Fig. 5C). Furthermore, Nutlin-3a-treated cells also showed increased expression of IL-6 (Fig. 5C). Simultaneously, ADSCs cultured with FN, and VN coatings showed a reduction in the expression of HMGB1 and secretion of IL-6 into a cytoplasm compared with the control. Moreover, the mRNA expression levels of SASP, *IL-1β, IL-6*, and *IL-10,* were determined (Supplementary Fig. S3). The results indicated a slight decrease of *IL-1β, IL-6,* and *IL-10* in ADSCs cultured with FN and VN coatings. Taken together, the senescent phenotype in ADSCs was regulated through AKT/MDM2/p53 pathway, in which ADSCs cultured with FN, VN exhibited the slow progression in this event.

## Discussion

Cellular aging is the progressive deterioration and functional decline of cells. This complex process is observed in vivo and *in vitro*^28,29^. Cell culture is the main process for cell processing performed using cells from autologous or allogeneic sources in cell therapy. Here, the challenge remains in the standardization of cell processing and ensuring the stability and yield of cell products in achieving optimal therapeutic doses^30^. In cell-based therapy, we found a certain number of clinical studies using ADSCs revealed a different clinical outcome, this variability might be from cellular components. Several factors can affect the cell's properties including essential nutrients, physio-chemical environment, substrate and surface properties, and cell density and confluence^31^. The quality of cell products is also a concern as the purity, potency, and proneness to senescence of MSCs used in cell therapy are critical^32^. Cellular senescence impairs replicative capacity and proliferation, affecting cell production. Senescent MSCs display phenotypic changes and release SASPs, including proinflammatory cytokines^33^. SASP can lead to chronic inflammation and affect neighboring cell behavior, proliferation, migration, and tissue regeneration capacity^34^. Therefore, the cell expansion process for cell therapy requires a good protocol to meet the high quality and quantity of MSCs.

FN and VN are extracellular matrix proteins widely used in cell culture aimed at promoting cell–matrix interaction. Cells with their specific integrin receptors can interact with FN and VN via the RGD sequence, which enhances cell attachment and promotes cell growth^22,23^. Additionally, other ECM proteins, like laminin and collagen also contribute significantly to cell adhesion and proliferation, showing similar effects on cell behavior when used as surface coatings. Notably, a previous study demonstrated growing bladder cancer cells in collagen cultured induced premature senescence via integrin β1/AKT^35^. Recent investigations showed the benefits of FN and VN in cell adhesion, resulting in a good option for use in clinical applications^36–38^. According to a recent study, by regulating the PI3K/AKT signaling pathway, FN and VN have the ability to modulate cell-cycle progression and prevent cellular senescence and apoptosis^24–27^. Moreover, binding of cells with FN and VN also influences the biological properties of cells through signaling pathways, such as activation of downstream signaling FAK–Src–PI3K^39–41^, AKT/mTOR pathway activation^40,41^, and MAPK/ERK pathway activation^42,43^. However, the roles of FN and VN in cell senescence have not been fully explored. Therefore, this study aimed to investigate the roles of FN and VN coatings in the senescence of ADSCs culture. The adhesion rate of ADSCs cultured with FN and VN coatings showed a significant increase. These coatings also contribute to an increased cell proliferation rate, as evidenced by the shorter PDT, which is frequently employed as a measure of proliferation capacity^44^. Shorter PDT represents the cell cycle for a population of cells to double in number through cell division. Culturing ADSCs with FN and VN coatings reduces cellular senescence, as evidenced by lower percentages of SA-β-gal positive cells at each passage compared to the control. Besides, the lower expression of senescence-associated markers, including *p16* and *p53-p21*, was reduced in ADSCs cultured with FN and VN coatings. The lower expression of these genes is associated with the attenuation of cellular senescence. Studies have also reported that the inactivation of *p16*^45,46^ and *p53-p21*^46,47^ signaling pathways can effectively inhibit cellular senescence.

The AKT/MDM2/p53 pathway is involved in cellular senescence and regulates the levels and activity of the p53 protein. AKT phosphorylates MDM2, leading to p53 degradation under normal conditions^48,49^. However, cellular stress inhibits AKT activity, resulting in reduced MDM2-mediated degradation of p53. Therefore, p53 can accumulate and induce senescence-associated cell-cycle arrest through p21. FN and VN coatings in ADSCs culture upregulated AKT expression and potentially enhanced cell survival and therapeutic efficacy. These coatings also influenced MDM2 regulation and reduced p53 expression, suggesting an impact on senescence in ADSCs. Overall, these results suggest that FN and VN coatings modulate AKT, MDM2, and p53 expressions and potentially regulate cellular senescence. In addition, FN-coated ADSCs upregulated α5 and β1 integrin genes, while VN-coated ADSCs upregulated αv, β3, and β5 integrin genes. This finding suggests that these integrins may play a role in cellular processes by activating AKT pathway. The increased expression of these integrin subunits may have implications for cell adhesion, migration, and survival.

Nutlin-3a, an MDM2 inhibitor^50^, was used to investigate the relationship between AKT, MDM2, and p53 in regulating cellular processes in ADSCs. Nutlin-3a functions by competitively inhibiting the binding of MDM2 to p53, preventing the ubiquitination and degradation of p53 which is leading to significantly increased p53 expression^50^. Nutlin-3a has been shown to induce cellular senescence through the MDM2-p53 pathway in various cell types, making it a valuable experimental model for studying the mechanisms underlying cellular senescence^51,52^. The results showed that the expression of AKT was reduced in ADSCs culture at passage 10 and increased in ADSC cultured with either FN or VN. Furthermore, ADSCs cultured with FN or VN also express integrin receptors such as α5 and αV, which can influence the activation of AKT^22,23^. Increased AKT activation may significantly enhance MDM2 activity, finally promoting the degradation of P53. Interestingly, ADSCs cultured with FN and VN coatings promoted AKT and MDM2 expressions while reducing p53 and p21 expressions. This observation suggests that the coatings may modulate these factors and impact cellular processes related to senescence and survival.

In addition, Nutlin-3a treatment increased the percentage of SA-β-gal positive cells, lead to cytoplasmic localization of HMGB1, and increased the expression of IL-6 in ADSCs culture. In contrast, ADSCs cultured with FN and VN coatings showed a lower percentage of SA-β-gal positive cells, reduced expression of HMGB1, and secretion of IL-6. These findings indicate that FN and VN coatings impact the cellular senescence markers and the expression of inflammatory molecules, such as HMGB1 and IL-6. Furthermore, various promising strategies rely on the prevention of proinflammatory-induced senescent cells. Recent studies are focused on the discovery of pharmacological agents that induce apoptosis in senescent cells. These substances are commonly referred to senolytic drugs or senolytics^53^. However, specific methodologies or treatment approaches for the cell culture process in this context have not been established.

This study demonstrated a promising procedure to cultivate ADSCs using FN and VN coatings. ADSCs cultured in these coatings exhibited improved cell adhesion, proliferation, and modulated senescence-associated markers. These results also suggest the potential role of FN and VN coatings in regulating cell senescence via AKT/MDM2/p53 pathway mediated by integrin α5 and αv. Our results showed culturing ADSCs with FN and VN coating is an easy and practical strategy that should be recommended as a good protocol for the preparation of ADSCs for therapeutic use. The impact of cellular senescence on the clinical outcomes of cell-based therapy can be significant. Therefore, further studies to investigate the impact of senescent cells on therapeutic outcomes should be performed. In addition, meta-analysis serves as a valuable tool in addressing this challenge and bridging the gap between laboratory studies and clinical applications.

## Methods

### Isolation and culture of ADSCs

The lipoaspiration technique was used to obtain adipose tissue samples from healthy subjects after their written informed consent. The samples were washed with warm phosphate-buffered saline (PBS) to eliminate blood and oil. Next, the adipose tissue was digested using 0.025% type I collagenase (Worthington Biochemical Corporation, Lakewood, NJ, USA) for 1 h at 37 °C. The lipoaspirate was centrifuged at 2000 *rpm* for 5 min to separate the pellet containing ADSCs, and the pellet was resuspended in stromal medium (DMEM-LG; Gibco, Carlsbad, CA, USA) supplemented with 10% fetal bovine serum (Sigma-Aldrich, St. Louis, MO, USA), 1% Penicillin–Streptomycin (Gibco, Carlsbad, CA, USA), and 1% Glutamax (Gibco, Carlsbad, CA, USA). The cells were seeded at a density of 10^3^ cells/cm^2^ and then incubated at 37 °C and 5% CO_2_. After 48 h, the nonadherent cells were removed, and the remaining cells were maintained until they reached 80%–90% confluence, while the medium was changed every three days. ADSCs were harvested using 0.25% trypsin–EDTA (Gibco, Carlsbad, CA, USA), and the cultures were either expanded or cryopreserved for future use.

### Surface modification and cell culture

To determine the effects of adhesion molecule as coating materials for ADSCs culture in vitro, the experiments were divided into three groups, ADSCs were cultured on uncoated surface as the control group (CTRL), coated surface with 5 µg/cm^2^ FN (Merck, Darmstadt, Germany) and 0.5 µg/cm^2^ VN (Advanced BioMatrix, Carlsbad, CA, USA). In the FN-coated group, 1 mL FN was added to a 35 mm dish, followed by the removal of excess solution. Air dried for at least 45 min at room temperature (RT) was performed. In the VN-coated group, 1 ml VN was added to 35 mm-dish, incubated for 1 h at RT, and then washed with PBS twice. To investigate the cellular senescence, 1 × 10^5^ ADSCs at the 2nd passage (P2) were seeded on uncoated (CTRL) and coated surface. ADSCs were cultured at 37 °C in 5% CO_2_ until the cells reached the 5th passage (P5), 7th passage (P7), and 10th passage (P10). The biological activities of ADSCs under each condition were evaluated at P5, P7, and P10. The MDM2 inhibitor, Nutlin-3a (Sigma-Aldrich, Taufkirchen, Germany), was dissolved in DMSO (PanReac Applichem, Darmstadt, Spain). Then 10 µM Nutlin-3a was added to the ADSCs culture at P10. This was followed by incubation for 48 h before concluding the experiment.

### Cell adhesion assay

1 × 10^5^ cells were seeded in a 24-well plate coated with FN and VN to evaluate the adhesion properties of ADSCs. Here, ADSCs cultured on an uncoated surface were used as control. The seeded cells were incubated for 12 h in a humidified atmosphere containing 5% CO_2_ at 37 °C, and then the nonadherent cells were carefully removed and discarded. The adherent cells were detached using 0.25% trypsin–EDTA (Sigma-Aldrich, St. Louis, MO, USA) and manually counted using a hemocytometer. The percentage of cell adhesion was calculated using the following equation to assess cell adhesion rate:

$${\text{Cell adhesion rate }}\left( \% \right) \, = \, \left( {{\text{Number of adherent cells}}/{\text{Number of seeding cells}}} \right) \times {1}00.$$

### Proliferative activity

ADSCs were seeded on control, FN, and VN-coated dishes at a density of 1 × 10^5^ cells to investigate the impact of adhesion molecule-coated surfaces on cell growth. Upon ADSCs cultured reaching 90% confluence, the cells were detached by trypsinization, and their viability was assessed using trypan blue staining. The proliferative activity of ADSCs was assessed by calculating the population doubling time (PDT) using the following expression: PDT = CT/PDN, where CT denotes the culture time in hours. PDN denotes the number of population doublings and is calculated using the formula: PDN = [logNH − logNI]/log2, where NI and NH represent the initial cell seeding number and the number of harvested cells, respectively. The PDT of ADSCs cultured on an uncoated surface was used as the control group for comparison.

### Senescence-associated β-galactosidase (SA-β-gal) staining

SA-β-gal activity was assessed using a senescence cell staining kit (Cell Signaling Technology, Danvers, MA, USA) to investigate the impact of coated surfaces of adhesion molecules on cellular senescence. Specifically, 3 × 10^4^ ADSCs at P5, P7, and P10 were seeded in 35-mm culture dishes with coated surfaces using FN and VN. Then, the cells were cultured in a humidified atmosphere containing 5% CO_2_ at 37 °C until the cultures reached 60% confluence. The cells were washed with PBS and then fixed for 15 min at RT before being stained with SA-β-gal staining solution in a dry incubator at 37 °C for 16–18 h. The formation of a blue color, which represented senescent-positive cells, was observed using an inverted microscope (Olympus, Shinjuku, Tokyo, Japan). The number of SA-β-gal-positive cells was counted and presented as the percentage of SA-β-gal activity using the following formula: SA-β-gal activity (%) = (Number of SA-β-gal-positive cells/Number of total cells) × 100.

### RNA extraction and quantitative real-time PCR

RNA was extracted using the phenol–chloroform procedure from the P5, P7, and P10 of ADSCs grown under individual conditions in TRIzol reagent (Invitrogen, Waltham, MA, USA). The RNA concentration was measured using a nanodrop spectrometer (Thermo Scientific, Waltham, MA, USA). cDNA was synthesized from 1 µg of RNA using iScript reverse transcription (Bio-Rad Laboratories, CA, USA), and the mRNA levels of *p16, p21, p53, ITGA5, ITGAV, ITGB1, ITGB3* and *ITGB5* were examined using quantitative real-time PCR. Each cDNA sample was mixed with a PCR master mix containing target gene forward and reverse primers (Table 1), nuclease-free water, and SYBR FAST qPCR master mix (KAPA Biosystem, Wilmington, MA, USA). Glyceraldehyde-phosphate dehydrogenase (*GAPDH*) was used as an internal reference control to normalize the expression levels of the genes of interest. Finally, the difference in transcript levels of senescence-associated genes was calculated using the comparative CT method (2^−∆∆Ct^) and presented as a relative gene expression to the control group.

**Table 1:** List of oligonucleotide primers for quantitative real-time PCR.

Gene	Forward primer sequences	Reverse primer sequences
*GAPDH*	5′-CAACTACATGGTTTACATGTTC CAA-3′	5′-CAGCCTTCTCCATGGTGGT-3′
*p16*	5′-TGAGGGTTTTCGTGGTTCAC-3′	5′-TGGTCTTCTAGGAAGCGGC-3′
*p21*	5′-GATGAGTTGGGAGGAGGCA G-3′	5′-CTGAGAGTCTCCAGGTCCAC-3′
*p53*	5′-ATGATTTGATGCTGTCCCCG-3′	5′-CAAGAAGCCCAGACGGAAAC-3′
*ITGA5*	5′-CTTCAACTTAGACGCGGAGG-3′	5′-GAGGTAGACAGCACCACCCT-3′
*ITGAV*	5′-CCGAAGCTCAGCCCTCTTG-3′	5′-GAAAAGCCATCGCCGAAGTG-3′
*ITGB1*	5′-GCCGCGCGGAAAAGATG-3′	5′-TGAATTTGTGCACCACCCAC-3′
*ITGB3*	5′-CCCATGAGTTGGCTGGGAAT-3′	5′-GCACTGTGGCCTCTCAGATT-3′
*ITGB5*	5′-AACTCGCGGAGGAGATGAG-3′	5′-GGTGCCGTGTAGGAGAAAGG-3′

### Western blotting analysis

For immunoblotting, the protein from ADSCs culture in different coated surfaces was dissolved in lysis buffer (Merck, Darmstadt, Germany) with protease inhibitors (Merck, Darmstadt, Germany), and the concentration was determined by Bicinchoninic acid (BCA) Protein Assay Kit (Thermo Scientific, Waltham, MA, USA). The 20 µg of protein samples were separated on a 10% polyacrylamide gel and transferred to an Immobilon-P transfer membrane (Merck Millipore, Burlington, MA, USA) using the Mini-Protean^©^ system (Bio-Rad Laboratories, Feldkirchen, Germany). The membranes were blocked in 5% skimmed milk (Merck, Darmstadt, Germany) for 2 h and incubated with the primary antibodies at 4 °C overnight. The primary antibodies used were AKT (#4691S), MDM2 (#86934S), p21 (#2947S), p53 (#2524S) (Cell Signaling Technology, Danvers, MA, USA), and β-Actin (Merck, Darmstadt, Germany) as loading controls for normalization. In this process, the membranes were cut before hybridization with various antibodies, resulting in the absence of images with adequate length.

On the following day, the membranes were incubated using an ECL Prime Western Blotting Detection Reagent (GE Healthcare, Buckinghamshire, UK) at RT for 2 h with secondary antibodies including anti-mouse IgG-HRP (#7074P2) and anti-rabbit IgG-HRP (#7076S) (Cell Signaling Technology, Danvers, MA, USA). The signal was then detected and analyzed via the ChemiDoc™ MP Imaging System (Bio-Rad Laboratories, Los Angeles, CA, USA).

### Immunofluorescent study

ADSCs samples were fixed in warm 4% paraformaldehyde for 15 min at RT and then washed three times with PBS. The samples were then permeabilized with 0.025% Triton X-100 (Sigma-Aldrich, St. Louis, MO, USA) for 10 min and blocked in 5% bovine serum albumin (BSA) (Sigma-Aldrich, St. Louis, MO, USA) in PBS for 1 h at RT. The samples were incubated overnight at 4 °C with primary antibodies, i.e., rabbit anti-HMGB1 (#3935S, 1:200) and rabbit IL-6 (#12912S, 1:200) (Cell Signaling Technology, Danvers, MA, USA), diluted in 1% BSA in PBS. The samples were then incubated with secondary antibody solutions, i.e., Alexa Fluor 488 goat antirabbit (AB150077, 1:250) (Abcam, Cambridge, UK), for 45 min at RT. Finally, the samples were counterstained with Prolong Gold™ Antifade Reagent with DAPI (Thermo Scientific, Waltham, MA, USA) for 24 h at RT in the dark. The samples were photographed using a fluorescence microscope (Olympus BX51, Shinjuku, Tokyo, Japan).

### Statistical analysis

The data are presented as mean ± standard deviation (SD) of at least three individual experiments (the Supplementary Figs. S1, and S3 are presented from two individual experiments). Statistical analysis was performed using an ordinary one-way ANOVA of GraphPad Prism 9.0 software (GraphPad Software, San Diego, CA, USA). A *p*-value < 0.05 was considered statistically significant.

### Statement

Human adipose tissue samples from healthy subject was approved by the Mahidol University Central Institutional Review Board and was performed in accordance with the Declaration of Helsinki (MU-CIRB 2018/202.1441) with the title “Study of Mesenchymal Stem Cells Senescence Derived Adipose Tissue”. There are no clinical trials or animal experiments in our research. All experiments were performed in accordance with relevant guidelines and regulations.

### Supplementary Information

Supplementary Information.

## Data Availability

The datasets generated during and/or analysed during the current study are available from the corresponding author on reasonable request.
