# Supplementary material for: Fibronectin and vitronectin alleviate adipose-derived stem cells senescence during long-term culture through the AKT/MDM2/P53 pathway
Source: Sci Rep. 2024 Jun 20;14:14242. doi: 10.1038/s41598-024-65339-z (PMC11189918; doi:10.1038/s41598-024-65339-z)
Supplement: Supplementary file 1 — Supplementary Information. [file 41598_2024_65339_MOESM1_ESM.docx]

**Supplementary**

**Table S1 The adhesion rate and population doubling time were evaluated in ADSC culture using FN and VN coatings at varying concentrations**

**
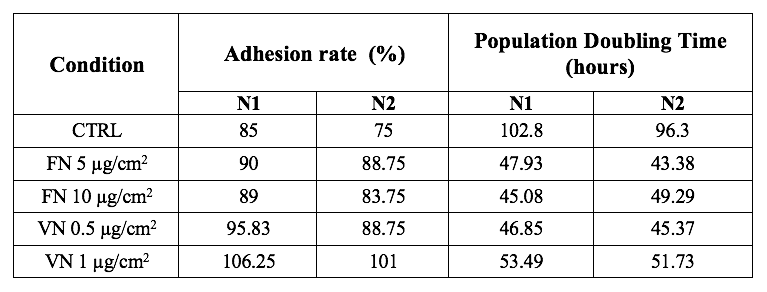
**

**Figure S1**


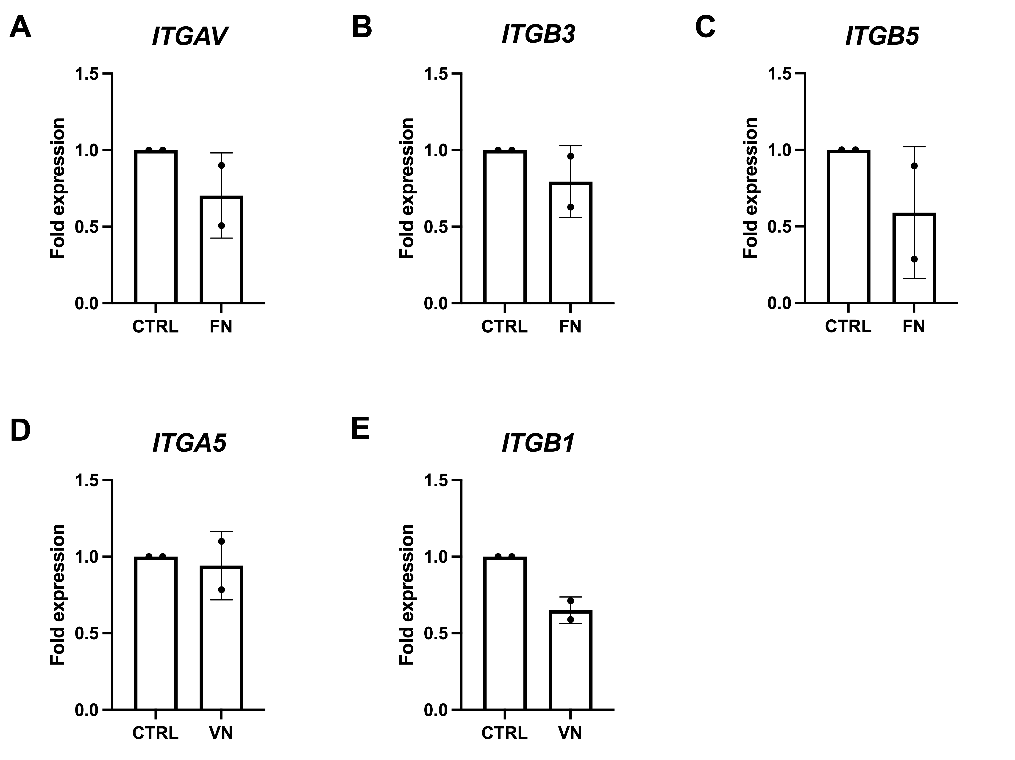


The expression levels of (A) *ITGAV,* (B) *ITGB3* and (C) *ITGB5* in ADSCs cultured with FN and (D) *ITGA5*, and (E) *ITGB1* in ADSCs cultured with VN. The data show the means ± SD from two independent experiments.

**Figure S2**


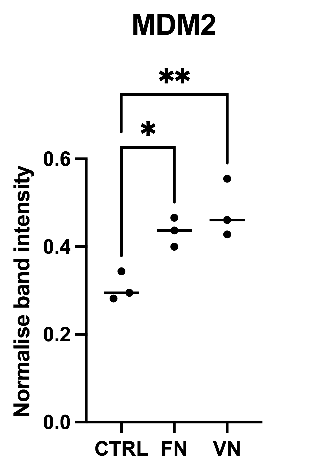


The expression of MDM2 between the CTRL, FN and VN (without Nutlin-3a) in P10. The result revealed significantly increased MDM2 expression in FN and VN coating. The data showed the means ± SD from three individual experiments. *p < 0.05 and **p < 0.01 vs. CTRL.

**Figure S3**


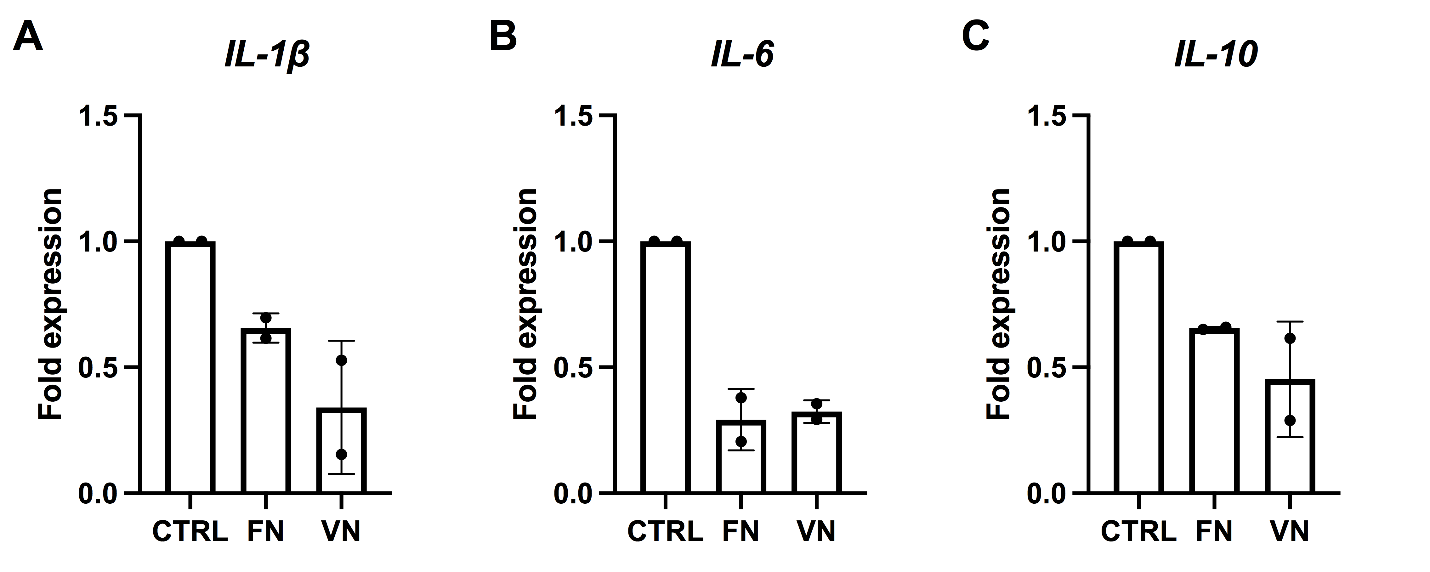


The mRNA expression levels of senescence-associated secretory phenotype (SASP) were performed, focusing on (A) IL-1β, (B) IL-6, and (C) IL-10. The presented data represent the means ± SD from two independent experiments (n = 2).

**Figure S4**


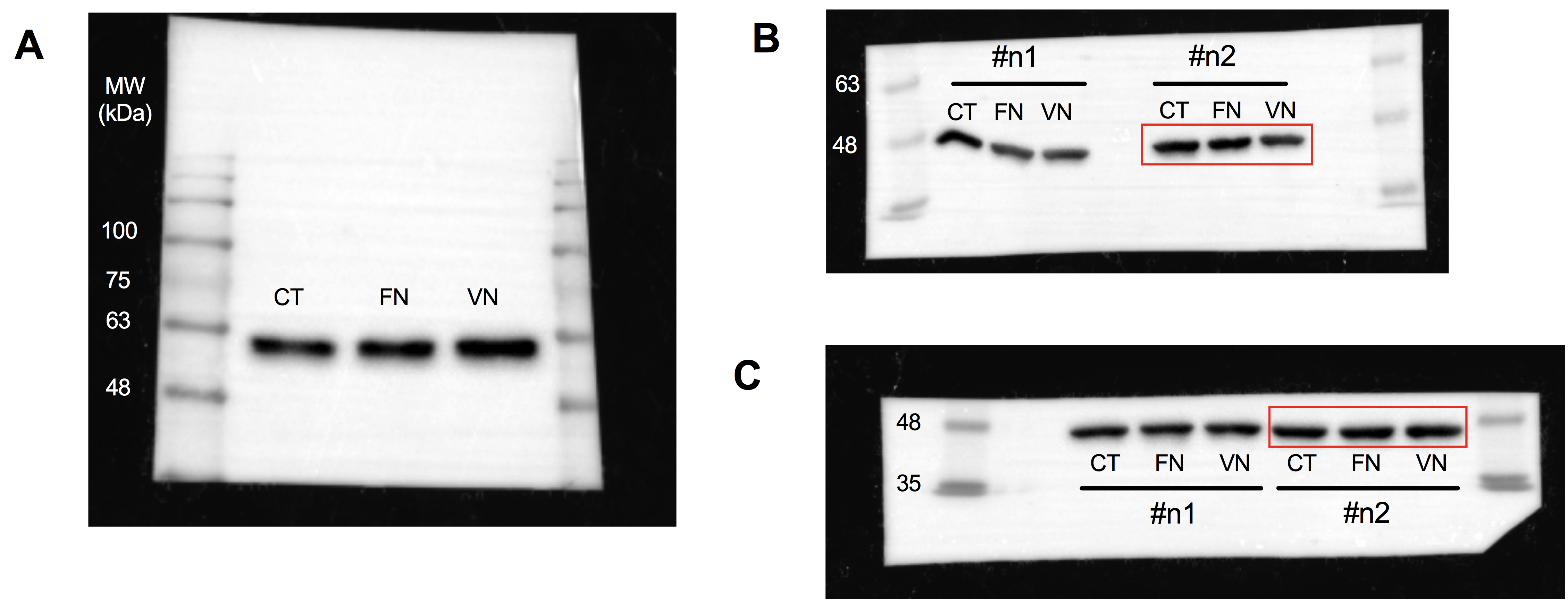


The presence of AKT-p53 signaling pathway at passage 5 detected by western blotting assay.
(A) AKT (60 kDa), (B) p53 (53 kDa) and (C) β-Actin (43 kDa). The red square indicating which lanes have been used to build figure in the research article.

**Figure S5**


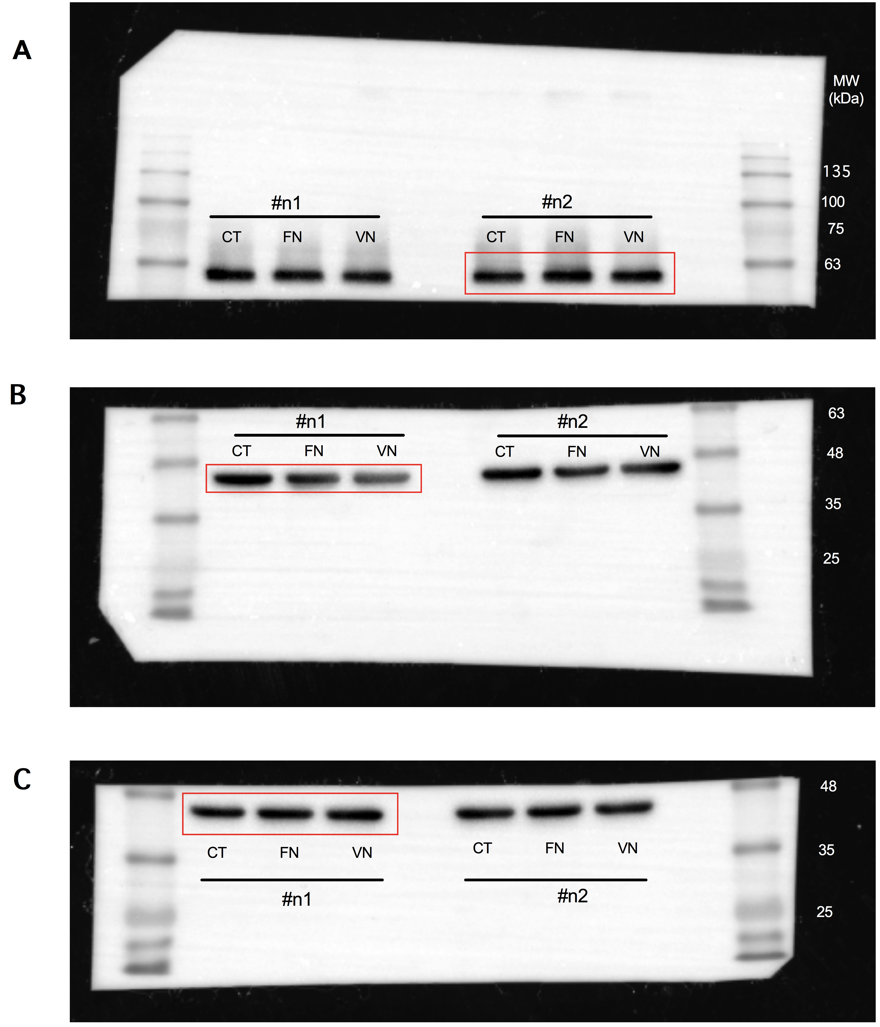


The presence of AKT-p53 signaling pathway at passage 7 detected by western blotting assay.
(A) AKT (60 kDa), (B) p53 (53 kDa) and (C) β-Actin (43 kDa). The red square indicating which lanes have been used to build figure in the research article.

**Figure S6**


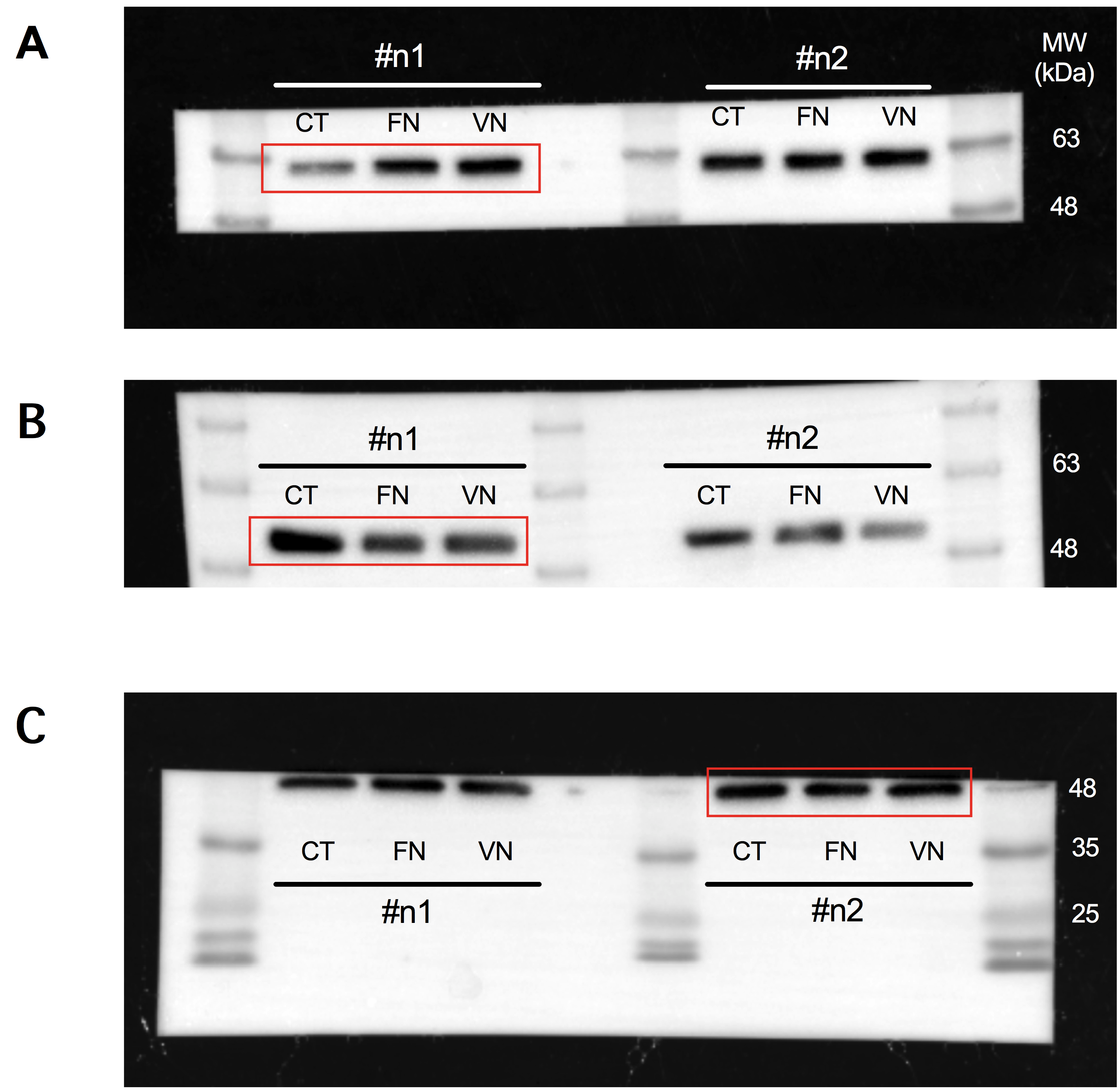


The presence of AKT-p53 signaling pathway at passage 10 detected by western blotting assay. (A) AKT (60 kDa), (B) p53 (53 kDa) and (C) β-Actin (43 kDa). The red square indicating which lanes have been used to build figure in the research article.

**Figure S7**

**
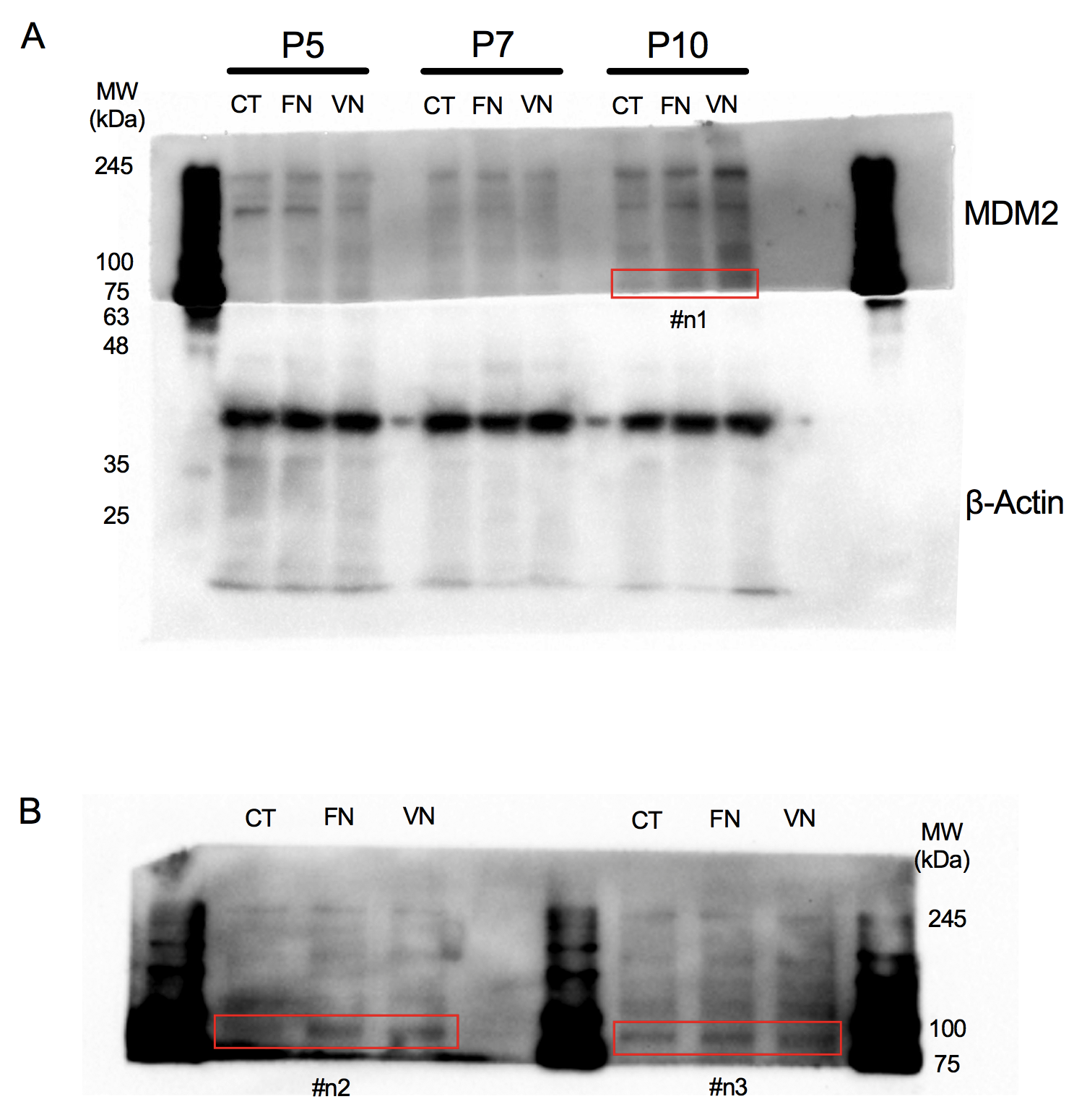
**

The presence of MDM2 (90 kDa) signaling pathway at passage 5, 7 and 10 detected by western blotting assay. (A) ADSCs from passages 5 and 7 do not detected band intensity on the blot, while cells from passage 10 show a visible band intensity. This outcome might indicate differences in protein expression or post-translational modifications between the passages, suggesting that the protein of interest is more prominently present in passage 10. (B) The presence of MDM2 (90 kDa) signaling at passage 10 detected by western blotting assay.
The red square indicates which lanes (n=3) were used for data analysis and the red square of #n1 used to build figure 3C.

.

**Figure S8**


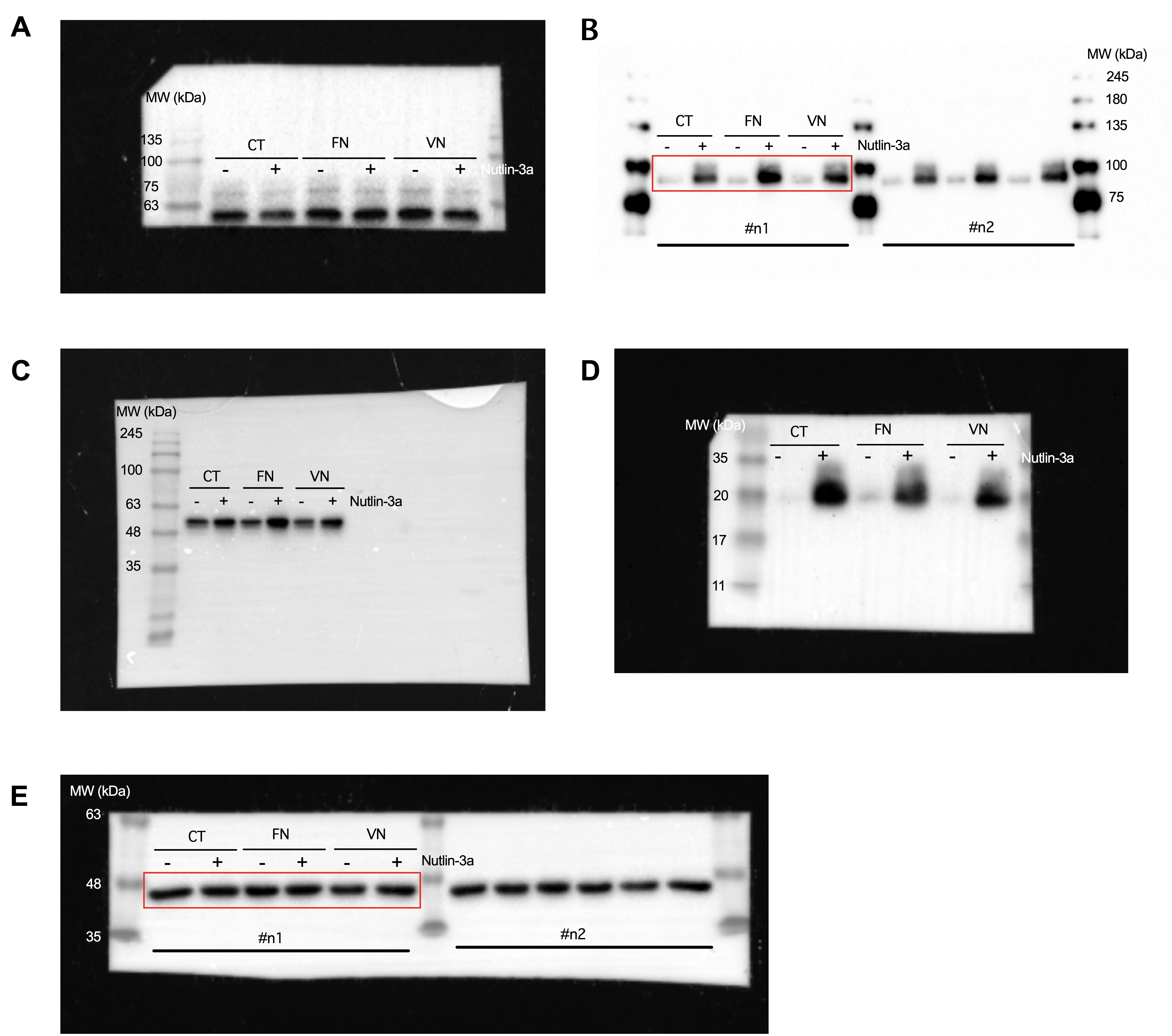


The regulation of cell senescence through AKT/MDM2/p53 signaling pathway at passage 10. ADSCs were cultured with and without FN and VN coatings, then treated with or without Nutlin-3a, and detected using western blotting assay. (A) AKT (60 kDa), (B) MDM2 (90 kDa), (C) p53 (53 kDa) (D) p21 (21 kDa), and
(E) β-Actin (43 kDa). The red square indicating which lanes have been used to build figure in the research article.
